# Barriers to clinical translation of small-diameter tissue-engineered vascular grafts in peripheral reconstruction

**DOI:** 10.1016/j.jvssci.2026.100435

**Published:** 2026-07-29

**Authors:** Miguel Castro e Silva, Leonor Baldaia, Luís Orelhas, Jorge Costa, Paula Dias, Beatriz Tavares, Eduardo Silva, Celso Nunes, Manuel Fonseca

**Affiliations:** Department of Angiology and Vascular Surgery, Unidade Local de Saúde de Coimbra, Coimbra, Portugal

**Keywords:** Tissue-engineered vascular graft, Small-diameter vascular graft, Peripheral arterial disease, Endothelialization, Biomaterials

## Abstract

**Objective:**

To provide a comprehensive review of small-diameter (<6 mm) tissue-engineered vascular grafts for peripheral vascular applications, examining mechanisms of graft failure, emerging biomaterial and fabrication strategies, and key considerations for clinical translation.

**Methods:**

A comprehensive literature review was performed using PubMed, Scopus, and Web of Science through January 2026. Studies were selected on the basis of relevance to peripheral vascular surgery, with emphasis on preclinical and clinical outcomes, biomaterial performance, fabrication approaches, and translational considerations, prioritizing recent studies while including seminal work.

**Results:**

Despite over four decades of research, small-diameter synthetic grafts continue to demonstrate poor patency in infrainguinal bypass, with expanded polytetrafluoroethylene and Dacron achieving 25% to 50% patency at 5 years compared with 60% to 80% for autologous saphenous vein in below-the-knee applications. Up to 20% of patients experience graft thrombosis within 6 months, with early failure associated with increased amputation rates. Graft failure is driven by acute thrombosis, intimal hyperplasia related to compliance mismatch and chronic inflammation, and incomplete endothelialization. Emerging strategies include multilayered scaffold architectures, hybrid biomaterials incorporating extracellular matrix components and bioactive molecules, advanced manufacturing techniques such as melt electrowriting, and immunomodulatory approaches promoting constructive remodeling. Recent preclinical studies report high patency rates approaching 100%, with complete endothelialization and minimal intimal hyperplasia in animal models. However, translation remains limited by inadequate study design, with median graft lengths of ∼5 cm and follow-up periods of ∼56 days, which do not reflect clinical scenarios where grafts often exceed 30 cm.

**Conclusions:**

Clinically viable small-diameter tissue-engineered vascular grafts for peripheral vascular reconstruction will require integrated optimization of hemocompatibility, endothelialization, biomechanical properties, and inflammatory response. Advances in biomaterials, manufacturing technologies, and mechanistic understanding suggest that grafts meeting clinical benchmarks may be achievable, but progress will depend on rigorous preclinical evaluation using clinically relevant models and end points.

The small-diameter vascular graft (<6 mm) represents a major unmet need in peripheral vascular surgery. Although large-diameter synthetic grafts achieve 5-year patency rates exceeding 95% for aortoiliac reconstruction, their small-diameter counterparts experience failure rates approaching 75% within 3 years for infrainguinal applications, rendering them a suboptimal choice for femoropopliteal and infrapopliteal bypass.^1^^,^^2^

The clinical burden of peripheral arterial disease (PAD) is substantial and growing. PAD affects over 200 million people worldwide, with chronic limb-threatening ischemia (CLTI) representing the most severe manifestation requiring surgical or endovascular intervention.^3^^,^^4^ For patients with CLTI, infrainguinal bypass remains a cornerstone of limb salvage, particularly when endovascular options are exhausted or anatomically unsuitable. The autologous great saphenous vein is the conduit of choice, achieving primary patency rates of 60% to 80% at 5 years for below-the-knee targets.^5^ However, 20% to 30% of patients lack suitable autologous conduits because of previous harvest for coronary or peripheral bypass, venous disease, varicosities, inadequate caliber, or anatomical limitations.^3^, ^4^, ^5^, ^6^

When autologous vein is unavailable, vascular surgeons face difficult choices. Synthetic grafts—primarily expanded polytetrafluoroethylene (ePTFE) and Dacron—demonstrate significantly inferior outcomes in small-diameter applications.

Beyond infrainguinal bypass, small-diameter grafts are also essential for arteriovenous access creation in patients requiring hemodialysis, representing a major and growing clinical need worldwide.^7^ When native arteriovenous fistula creation is not feasible, prosthetic arteriovenous grafts provide an alternative, although with inferior patency and higher intervention rates compared with native fistulae.^7^ A randomized trial comparing 6 vs 6 to 8 mm tapered grafts for brachial-axillary dialysis access demonstrated significantly better primary patency (85% vs 62% at 1 year) and lower complication rates with larger-diameter grafts, further illustrating the diameter-dependent nature of graft outcomes.^8^

Additional applications include reconstruction after traumatic vascular injury, visceral and renal artery bypass, and upper-extremity revascularization, where the lack of reliable small-diameter grafts continues to limit surgical options.

This review examines the mechanisms underlying small-diameter graft failure, evaluates emerging biomaterial and fabrication strategies, and highlights key challenges in preclinical design and clinical translation for peripheral vascular applications.

## Methods

A comprehensive literature review was performed to evaluate the current state of small-diameter (<6 mm) tissue-engineered vascular grafts (TEVGs) for peripheral vascular applications. A search of PubMed, Scopus, and Web of Science was conducted through January 2026 using combinations of the following terms: “small-diameter vascular graft,” “tissue-engineered blood vessel,” “peripheral arterial bypass,” “infrainguinal reconstruction,” and “vascular access graft.”

Studies were selected on the basis of their relevance to peripheral vascular surgery and clinical translation. Both preclinical and clinical studies were considered, with particular emphasis on graft performance, mechanisms of failure, biomaterial characteristics, fabrication techniques, and translational applicability. Priority was given to recent publications, whereas seminal studies were included to provide context and foundational understanding.

The included literature was analyzed qualitatively, with synthesis focused on key mechanisms of graft failure, emerging engineering strategies, and factors influencing successful translation to clinical practice.

## Mechanisms of small-diameter graft failure

**Fig 1:**
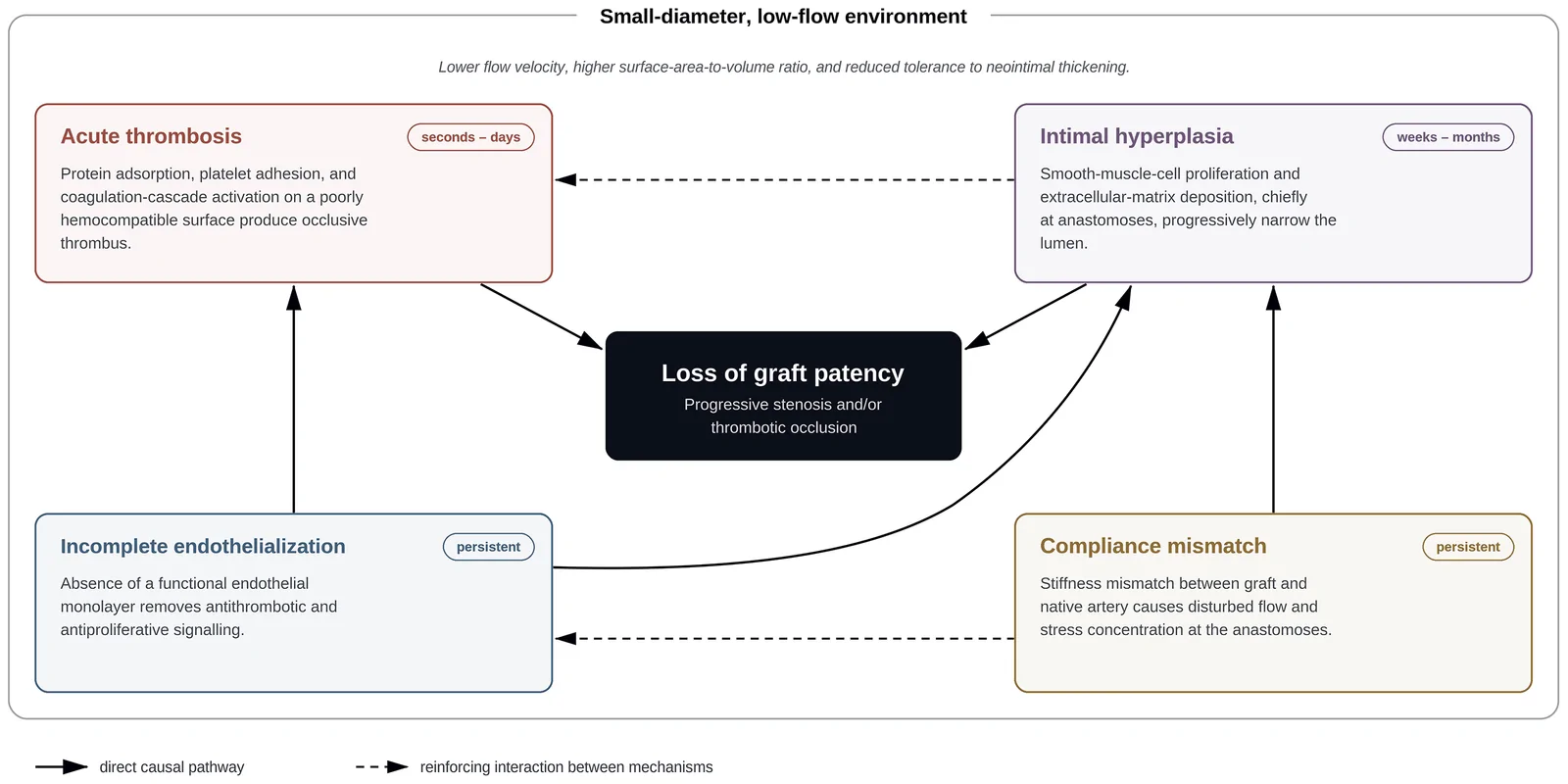
Interconnected mechanisms of small-diameter vascular graft failure. Schematic representation of the four principal mechanisms of failure—acute thrombosis, intimal hyperplasia, incomplete endothelialization, and compliance mismatch—shown within the small-diameter, low-flow environment that potentiates them. *Solid arrows* denote direct causal pathways; *dashed arrows* denote reinforcing interactions between mechanisms. Together these processes culminate in progressive luminal stenosis and thrombotic occlusion.

Understanding why small-diameter grafts fail is essential for developing effective solutions. Failure occurs through four interconnected mechanisms: acute thrombosis, intimal hyperplasia (IH), compliance mismatch, and inadequate endothelialization.^9^^,^^10^ These mechanisms act across different time scales but must be addressed simultaneously. These mechanisms and their corresponding engineering strategies are summarized in Table I. These four mechanisms and their temporal interplay are depicted schematically in Fig 1.

**Table I:** Mechanisms of small-diameter graft failure and corresponding engineering strategies

Mechanism	Pathophysiology	Design challenge	Engineering strategies
Acute thrombosis	Platelet adhesion and coagulation cascade activation due to poor hemocompatibility	Prevent early graft occlusion	Antithrombogenic surface modification (heparin and nitric oxide donors), endothelialization strategies
Intimal hyperplasia	Smooth muscle cell proliferation driven by compliance mismatch and chronic inflammation	Prevent late stenosis	Compliance matching, bioactive molecule delivery (eg, VEGF and CNP), multilayered scaffold design
Compliance mismatch	Mechanical mismatch between graft and native artery leading to disturbed flow and altered wall stress	Maintain physiological hemodynamics	Biomaterial selection, structural optimization, precision fabrication (eg, melt electrowriting)
Incomplete endothelialization	Lack of functional endothelial lining leading to thrombosis and inflammation	Promote rapid endothelial coverage	Extracellular matrix-based materials, surface functionalization, cell seeding approaches

*CNP*, C-type natriuretic peptide; *VEGF*, vascular endothelial growth factor.

### The diameter-patency relationship

Graft diameter is the single most important predictor of patency. A systematic review of 92 studies confirmed that smaller diameter grafts have significantly higher rates of thrombosis and nonpatency, with the relationship being exponential rather than linear—as diameter decreases below 6 mm, failure rates accelerate dramatically.^11^

The physical basis for this relationship involves multiple interacting factors.^2^ Lower flow velocities in smaller grafts increase residence time for coagulation cascade activation, allowing thrombus formation to proceed unchecked. Higher wall shear stress activates platelets and damages endothelial cells on the graft surface. In addition, a greater surface area-to-volume ratio amplifies the impact of thrombogenic surfaces, as a larger proportion of blood volume contacts the foreign material. Finally, reduced tolerance for intimal thickening means that even modest neointimal formation – clinically insignificant in larger grafts – can cause critical stenosis in small vessels.^2^, ^3^, ^4^, ^5^, ^6^, ^7^^,^^9^^,^^12^^,^^13^

### Acute thrombosis: The immediate challenge

Acute thrombosis represents the most immediate failure mode for small-diameter grafts, occurring when the blood-contacting surface fails to resist platelet adhesion and coagulation cascade activation.^5^, ^6^, ^7^^,^^9^^,^^10^^,^^12^^,^^13^ In peripheral vascular applications, this failure mode is particularly consequential: up to 20% of patients experience graft thrombosis within 6 months of lower-extremity bypass surgery, with early thrombosis associated with significantly higher amputation rates.^12^

The thrombotic cascade on synthetic surfaces follows a predictable sequence.^5^ Protein adsorption occurs within seconds, as fibrinogen, von Willebrand factor, and other plasma proteins adsorb to the graft surface, creating a conditioning film. Platelet adhesion follows within minutes, as platelets bind to adsorbed proteins via glycoprotein receptors (GPIIb/IIIa and GPIb). Platelet activation proceeds over minutes to hours, with adhered platelets releasing prothrombotic mediators including adenosine diphosphate, thromboxane A_2_, and serotonin. Finally, thrombus propagation occurs over hours to days, as activated platelets recruit additional platelets and trigger the coagulation cascade, forming occlusive thrombus.

Passive antithrombogenic strategies aim to minimize protein adsorption and platelet adhesion through surface modifications. A comprehensive 2025 review categorized these approaches based on physicochemical mechanisms.^5^

Hydrophilic polymer coatings create a hydration layer that resists protein adsorption. poly(ethylene glycol) (PEG) represents the classical approach, creating steric repulsion through hydrated polymer chains. However, PEG suffers from oxidative degradation in vivo, limiting long-term efficacy.^5^ Zwitterionic polymers – including phosphorylcholine, sulfobetaine, and carboxybetaine – demonstrate superior stability through electrostatic interactions and show promise in recent studies, though they require covalent grafting for durability.^5^ Poly(2-oxazoline)s represent an emerging alternative to PEG with tunable properties and improved stability.^5^

Heparin immobilization remains the most clinically validated surface modification approach.^14^, ^15^, ^16^ Covalent heparin grafting provides sustained anticoagulant activity by catalyzing antithrombin III-mediated inactivation of thrombin and factor Xa. Advanced functionalization strategies combining heparin with additional bioactive molecules show particular promise for peripheral vascular applications.

A heparin-aspirin compound combining anticoagulation with antiplatelet effects achieved complete patency for 1 month in rat models, whereas unmodified controls showed severe obstruction within 1 week.^14^ Heparin/arginine-glycine-aspartic acid coimmobilization on PTFE simultaneously reduced platelet adhesion and promoted endothelial cell attachment, addressing both acute thrombosis and long-term endothelialization.^15^ Triple functionalization with heparin, stromal cell-derived factor-1α, and cluster of differentiation 47 achieved simultaneous antithrombogenicity, endothelial progenitor cell (EPC) recruitment, and anti-inflammatory effects through a versatile plasma immersion ion implantation approach.^16^

Clinical anticoagulation strategies reflect the inadequacy of surface modifications alone. A 2024 Journal of the American College of Cardiology Scientific Statement on antithrombotic strategies for patients with PAD noted that dual antiplatelet therapy has not been shown to reduce graft failure, reintervention, or amputation in lower-extremity bypass.^12^ Full-dose anticoagulation with warfarin or direct oral anticoagulants is commonly prescribed by vascular surgeons, particularly for prosthetic grafts to below-the-knee targets.^12^ The optimal antithrombotic strategy must consider bleeding risk, conduit type, bypass target, and distal outflow quality.^12^ The fact that systemic anticoagulation remains standard practice despite surface modifications underscores the severity of the thrombosis challenge and the inadequacy of current solutions.

### Intimal hyperplasia: The chronic challenge

IH represents the primary cause of late graft failure, characterized by excessive smooth muscle cell (SMC) proliferation and extracellular matrix deposition that progressively narrows the lumen.^9^^,^^10^ In peripheral bypass grafts, IH develops preferentially at anastomotic sites, creating hemodynamically significant stenoses that compromise limb perfusion.

The mechanisms of IH development involve complex interplay of mechanical, inflammatory, and cellular factors.^9^^,^^10^ Compliance mismatch between graft and native artery creates flow disturbances and oscillating shear stress at anastomoses, stimulating SMC proliferation.^2^, ^3^, ^4^, ^5^, ^6^, ^7^^,^^9^^,^^12^^,^^13^ Inflammatory activation from foreign body response to synthetic materials drives macrophage infiltration and release of growth factors – including platelet-derived growth factor, transforming growth factor-β, and fibroblast growth factor – that stimulate SMC proliferation.^8^^,^^10^^,^^11^^,^^14^, ^15^, ^16^, ^17^, ^18^ Endothelial dysfunction or absence eliminates normal antiproliferative signals [nitric oxide (NO) and prostacyclin], permitting unchecked SMC growth.^8^^,^^10^^,^^11^^,^^14^, ^15^, ^16^, ^17^ Mechanical injury during anastomosis and suturing triggers wound healing responses that contribute to neointimal formation.^2^, ^3^, ^4^, ^5^, ^6^, ^7^^,^^9^^,^^12^^,^^13^

Importantly, the specific causes of IH have not been fully deduced, but thrombosis formation due to bioincompatibility and biomechanical property mismatch are attributed to its initiation.^9^ This mechanistic uncertainty complicates rational intervention design but also suggests that addressing thrombosis and compliance mismatch may simultaneously reduce IH.

Strategies to combat IH focus on biomimetic multilayered structures that replicate native vessel architecture and function.^8^, ^9^, ^10^, ^11^^,^^14^, ^15^, ^16^, ^17^, ^18^, ^19^, ^20^ A core-shell fibrous graft fabricated by coaxial electrospinning achieved this architecture: the shell [polycaprolactone (PCL)/gelatin loaded with heparin-vascular endothelial growth factor (VEGF)] provided rapid degradation to release bioactive molecules, whereas the core (pure PCL) maintained mechanical strength.^19^ After 4 months in rat aorta, this graft demonstrated 100% patency with complete endothelialization and smooth muscle regeneration without IH.^19^

Cardiac extracellular matrix-based bilayer grafts represent another innovative approach.^20^ Liquid chromatography-tandem mass spectrometry proteome analysis revealed that cardiac extracellular matrix is enriched with bioactive proteins related to angiogenesis, wound regeneration, and cell migration.^20^ When combined with a heparinized inner layer and superporous outer layer, these grafts achieved 100% patency at 10 weeks with complete endothelial lining and no IH.^20^ Ribonucleic acid sequencing confirmed that hypoxia-inducible factor 1 and VEGF signaling pathways drove endothelial remodeling.^20^

NO release strategies address both thrombosis and IH.^17^ *S*-nitrosated keratin nanoparticles electrospun with PCL provided prolonged NO release for up to 25 days in the presence of physiologically reducing agents.^17^ Combined with resveratrol in a bilayer design, these grafts promoted endothelial proliferation while inhibiting excessive SMC proliferation, achieving 100% patency at 3 months in rat abdominal aorta with accelerated endothelialization and no IH, thrombosis, or calcification.^17^

Immunomodulation through macrophage polarization has emerged as a critical strategy.^8^^,^^10^^,^^11^^,^^14^, ^15^, ^16^, ^17^, ^18^ VEGF local administration induced M2 macrophage polarization, reducing proinflammatory interleukin-1 and increasing anti-inflammatory interleukin-10, resulting in thinner neointima and fibrous capsule.^18^ M2-polarized macrophages secrete factors that promote endothelialization while suppressing excessive SMC proliferation.^8^^,^^10^^,^^11^^,^^14^, ^15^, ^16^, ^17^, ^18^ This approach addresses the root inflammatory cause of IH rather than merely treating symptoms.

### The endothelialization imperative

Rapid and complete endothelialization is universally recognized as the key to preventing both thrombosis and IH.^4^, ^5^, ^6^, ^7^, ^8^, ^9^, ^10^, ^11^, ^12^, ^13^, ^14^, ^15^, ^16^, ^17^, ^18^, ^19^, ^20^, ^21^ A functional endothelial monolayer provides an antithrombogenic surface through heparan sulfate proteoglycans, thrombomodulin, and tissue plasminogen activator; NO production that inhibits platelet activation and SMC proliferation; barrier function preventing inflammatory cell infiltration and SMC migration; and mechanotransduction that regulates vascular tone and remodeling in response to flow.^10^

Different endothelialization strategies are required depending on the clinical context and graft characteristics.^21^

In vitro preseeding with endothelial cells has been studied extensively. Autologous endothelial cells harvested from patient vessels provide optimal biocompatibility but require invasive harvest and extended culture time.^8^, ^9^, ^10^, ^11^^,^^13^, ^14^, ^15^, ^16^, ^17^, ^18^, ^19^, ^20^, ^21^ EPCs from peripheral blood offer less invasive sourcing but variable differentiation capacity.^21^ Induced pluripotent stem cell-derived endothelial cells provide unlimited supply and can be premanufactured, but face regulatory complexity.^21^

Early clinical studies demonstrated that endothelial cell-seeded 4-mm Dacron grafts achieved higher patency rates than nonseeded grafts or PTFE grafts, with endothelium present to a significant extent only on the seeded grafts.^13^ However, preseeding requires weeks of culture and specialized facilities, limiting clinical practicality for urgent peripheral vascular interventions.^21^

In situ endothelialization through surface modifications that capture circulating EPCs offers a more practical approach for peripheral vascular applications. Anti-CD34 antibody functionalization directly captures EPCs from blood flow, accelerating endothelialization without preseeding.^16^, ^17^, ^18^, ^19^, ^20^, ^21^, ^22^ Stromal cell-derived factor-1α immobilization creates chemotactic gradients that recruit EPCs to the graft surface.^16^ VEGF incorporation stimulates EPC proliferation and differentiation into mature endothelial cells.^8^^,^^10^^,^^11^^,^^14^, ^15^, ^16^, ^17^, ^18^, ^19^

### Biomechanical requirements and compliance matching

Mechanical property mismatch between synthetic grafts and native arteries is a fundamental contributor to failure through multiple mechanisms.^1^, ^2^, ^3^, ^4^, ^5^, ^6^, ^7^^,^^9^^,^^12^^,^^13^ Native peripheral arteries exhibit specific biomechanical properties: burst pressure of 1680 to 3000 mmHg, compliance of 3% to 7% per 100 mmHg for muscular arteries and 10% to 15% for elastic arteries, suture retention strength exceeding 2 N for safe anastomosis, and elastic modulus of 0.5 to 2 MPa in physiological range.^2^, ^3^, ^4^, ^5^, ^6^, ^7^, ^8^, ^9^, ^10^, ^11^, ^12^, ^13^, ^14^, ^15^, ^16^, ^17^, ^18^, ^19^, ^20^, ^21^, ^22^, ^23^, ^24^

When graft compliance differs from native artery, several pathological processes occur.^2^, ^3^, ^4^, ^5^, ^6^, ^7^^,^^9^^,^^12^^,^^13^ Flow disturbances at anastomoses create regions of oscillating shear stress and flow separation. Stress concentration at the junction between compliant artery and stiff graft damages endothelium. Altered mechanotransduction disrupts normal endothelial and SMC responses to hemodynamic forces. IH develops preferentially in regions of disturbed flow and abnormal stress. Clinical evidence suggests that patency rates improve when graft elastic properties match the native artery.^2^

Biomimetic approaches to compliance matching have achieved significant advances. Melt electrowriting (MEW) with sinusoidal fiber architecture achieved a breakthrough: wave-like circumferential fibers mimicking collagen fiber arrangement in native arteries exhibited physiological compliance of 12.9% ± 0.6% per 100 mmHg, whereas straight-fiber controls showed only 5.5% ± 0.1% compliance.^23^ Burst pressures reached 1078 ± 236 mmHg for optimized designs, and fiber diameters of 15 ± 2 μm enabled high architectural precision and reproducibility.^23^ This represents a paradigm shift: rather than simply selecting compliant materials, architectural design at the microscale can engineer compliance into otherwise stiff polymers.^23^

Completely biological scaffoldless grafts offer inherent compliance matching.^25^ Human mesenchymal stem cell-based grafts with highly aligned ECM nanofibers demonstrated anisotropic mechanical properties similar to native vessels.^25^ Completely biological composition eliminates foreign body response and enables full remodeling, though manufacturing complexity and regulatory considerations remain challenging.^25^

Hybrid approaches combining synthetic strength with biological compliance show particular promise for peripheral vascular applications.^19^, ^20^, ^21^, ^22^, ^23^, ^24^ Thermoplastic polyurethane/silk fibroin nanofibers with chitosan hydrogel coating achieved elastic modulus of 4.92 ± 0.11 MPa, burst pressure of 1140 ± 12 mmHg, and elongation at break of 196% ± 4%.^24^ Cardiac ECM/PCL bilayer designs with controlled microstructures provided mechanical strength while maintaining biological functionality.^20^ Core-shell structured micronanofibers separated mechanical (core) from biological (shell) functions, enabling independent optimization of conflicting requirements.^19^

## Biomaterial strategies and comparative performance

**Fig 2:**
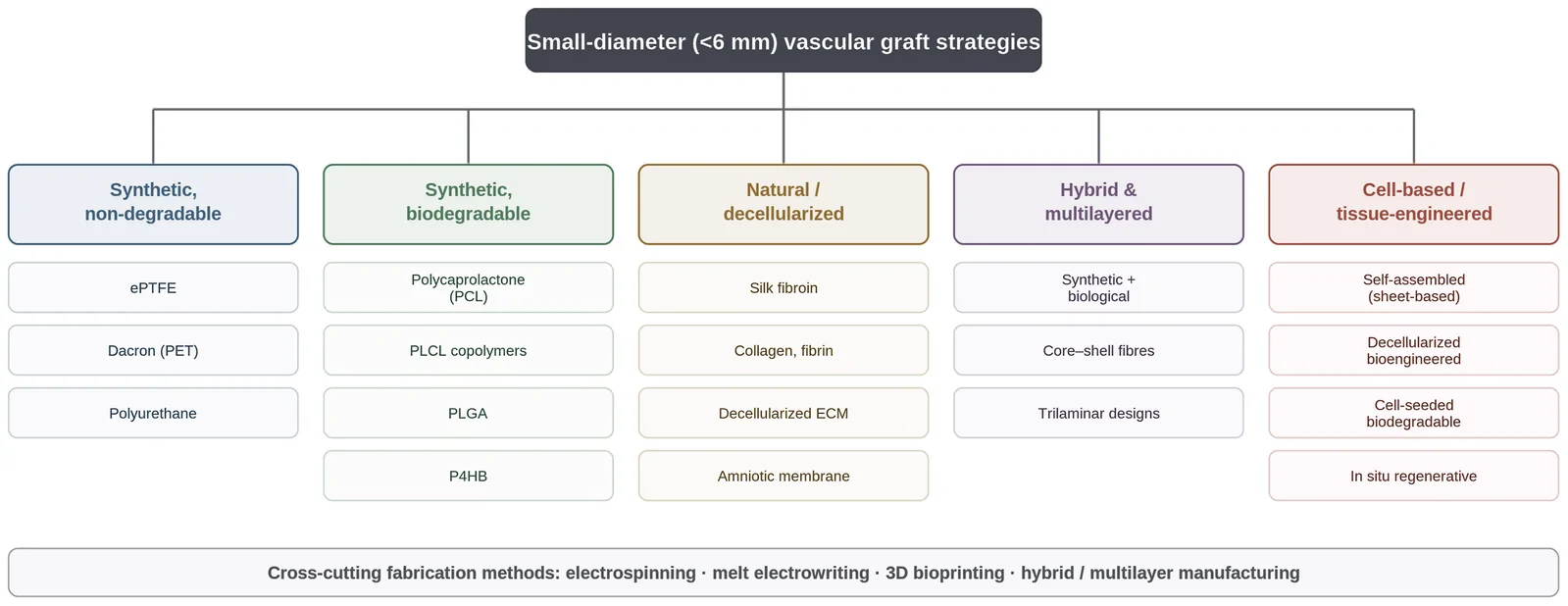
Classification of biomaterial and conduit strategies for small-diameter vascular grafts. Overview of the principal construct categories, from synthetic (nondegradable and biodegradable) and natural or decellularized biomaterials to hybrid, multilayered, and cell-based tissue-engineered vessels, with representative examples of each approach. *3D*, Three-dimensional; *ECM*, Extracellular matrix; *ePTFE*, expanded polytetrafluoroethylene; *P4HB*, poly-4-hydroxybutyrate; *PCL*, polycaprolactone; *PET*, polyethylene terephthalate; *PLCL*, poly(L-lactide-co-ε-caprolactone); *PLGA*, poly(lactic-co-glycolic acid).

No single material achieves all required properties simultaneously, driving the field toward hybrid approaches combining advantages of multiple material classes. Understanding the comparative performance of different biomaterials is essential for rational graft design. The principal biomaterial and conduit strategies discussed below are classified in Fig 2.

### Synthetic biodegradable polymers

PCL has emerged as the most extensively studied biodegradable polymer for small-diameter TEVGs due to favorable mechanical properties, processability, and US Food and Drug Administration approval for other medical applications.^26^, ^27^, ^28^

Advantages of PCL include excellent structural integrity with no aneurysmal dilation over 18 to 24 months in animal models; superior endothelialization compared with ePTFE with significantly better endothelial coverage at all time points; better healing characteristics including enhanced macrophage and fibroblast ingrowth, extracellular matrix formation, and neoangiogenesis; 100% patency in rat models at 24 weeks compared with stenotic lesions in 40% to 50% of ePTFE controls; and tunable degradation through molecular weight and fiber architecture modifications.^26^, ^27^, ^28^

However, PCL presents significant limitations. Slow biodegradation results in scaffolds retaining fibrillar structure even at 180 days, limiting complete tissue replacement.^27^, ^28^, ^29^ More concerning, calcification appears universal across PCL studies, with chondroid metaplasia appearing in neointima after 12 weeks and progressing to calcium phosphate deposits by 180 days.^27^, ^28^, ^29^ This represents a significant barrier to clinical translation for peripheral vascular applications, as calcification leads to graft stiffening, compliance mismatch, and eventual failure.

Strategies to overcome PCL limitations include heparin immobilization via gamma irradiation and N-(3-dimethylaminopropyl)-N′-ethyl carbodiimide hydrochloride/ N-hydroxysuccinimide chemistry to improve antithrombogenicity; VEGF loading to promote endothelial cell proliferation and prevent thrombosis; C-type natriuretic peptide incorporation which improved hemocompatibility, promoted M2 macrophage polarization, and enhanced contractile SMC regeneration; and blending with natural polymers (such as gelatin, collagen, and chitosan) to improve biocompatibility while maintaining mechanical strength.^30^^,^^31^

Poly(L-lactide-co-ε-caprolactone) (PLCL) copolymers offer tunable degradation rates and mechanical properties by adjusting the lactide/caprolactone ratio.^32^ When combined with recombinant humanized type III collagen and crosslinked with procyanidins, PLCL grafts demonstrated good cell and blood compatibility with anticalcification performance, 30-day patency in rabbit carotid artery transplantation, and inhibitory effects on SMC proliferation and IH.^32^ The use of procyanidins as a natural crosslinker – replacing cytotoxic glutaraldehyde – represents an important advance for clinical translation.^32^

Polyurethanes offer superior compliance matching compared with other synthetic polymers. A poly(ether)urethane-polydimethylsiloxane semi-interpenetrating polymeric network demonstrated superior handling and compliance characteristics compared with ePTFE, higher patency rates in sheep carotid artery model, in vivo remodeling with gradual replacement by natural tissue, and no calcification over the study period.^7^ The dual-layer architecture with different porous layers in wall thickness enabled both mechanical stability and cellular infiltration.^7^

### Natural and hybrid biomaterials

Silk fibroin from Bombyx mori has emerged as a leading natural biomaterial for small-diameter TEVGs, with over 30 years of development in vascular tissue engineering.^33^, ^34^, ^35^, ^36^

Key advantages of silk fibroin include excellent long-term patency—85.1% at 1 year vs 30% for PTFE in rat abdominal aorta model, antithrombotic surface providing inherent resistance to platelet adhesion, favorable biodegradation with collagen content increasing as fibroin content decreases over time, bone marrow-derived cell recruitment contributing to vascular remodeling, tailorable mechanical properties through processing modifications, and minimal inflammatory reactions compared to synthetic alternatives.^33^, ^34^, ^35^, ^36^

Three-layered silk fibroin vascular scaffolds (SILKGraft) achieved compliance values approaching those of saphenous and umbilical veins through optimized textile layer arrangement.^37^ The corrugation approach improved kink resistance while maintaining biocompatibility – a critical consideration for infrainguinal bypass where grafts traverse the knee joint.^37^

Decellularized extracellular matrix from various sources provides native biological cues that synthetic materials cannot replicate.^4^, ^5^, ^6^, ^7^, ^8^, ^9^, ^10^, ^11^, ^12^, ^13^, ^14^, ^15^, ^16^, ^17^, ^18^, ^19^, ^20^, ^21^, ^22^, ^23^, ^24^, ^25^, ^26^, ^27^, ^28^, ^29^, ^30^, ^31^, ^32^, ^33^, ^34^, ^35^, ^36^, ^37^, ^38^, ^39^, ^40^

Bovine pericardium decellularized extracellular matrix combined with poly(propylene fumarate) created biohybrid scaffolds with reduced donor variability through decellularization, modulus matched to human coronary arteries and saphenous veins, and demonstrated re-endothelialization and tissue growth in vivo.^40^

Human amniotic membrane provided bioactive milieu for rapid endothelial cell proliferation, resisted fibroblast-induced collagen secretion reducing IH, maintained patency by progressively stabilizing remodeling structure over 24 weeks, and demonstrated less intensive ECM deposition and weaker inflammatory response compared with porcine small intestinal submucosa.^41^

### Multilayered architectures: the emerging standard

The most successful recent approaches employ multilayered designs where each layer addresses specific requirements – a strategy analogous to the native vessel's trilaminar architecture.^8^, ^9^, ^10^, ^11^^,^^14^, ^15^, ^16^, ^17^, ^18^, ^19^, ^20^

The inner layer provides dense, smooth, and antithrombogenic surface promoting rapid endothelialization. The middle layer contributes mechanically robust structure providing burst strength and compliance. The outer layer offers porous scaffold supporting SMC infiltration and adventitial integration.

## Fabrication methods for clinical translation

The choice of fabrication method significantly impacts graft properties, manufacturing scalability, and regulatory pathway. For peripheral vascular applications requiring off-the-shelf availability and consistent performance, manufacturing considerations are paramount.

## Electrospinning: The dominant technology

Electrospinning remains the most widely used fabrication method for small-diameter TEVGs due to its ability to create nanofibrous structures mimicking native ECM architecture.^1^

Advantages include nanoscale fiber production (50-500 nm) mimicking native ECM collagen fibrils; high surface area-to-volume ratio promoting cell adhesion and proliferation; tunable porosity and fiber alignment through process parameter control; compatibility with diverse polymers including PCL, PLCL, polylactic acid, silk, collagen, and blends; and scalable manufacturing with established industrial equipment.^1^

Limitations include restricted control over three-dimensional (3D) architecture compared to additive manufacturing; difficulty achieving complex geometries for patient-specific applications; potential for solvent residues affecting biocompatibility; and fiber fusion at high humidity reducing porosity.^1^, ^2^, ^3^, ^4^, ^5^, ^6^, ^7^, ^8^, ^9^, ^10^, ^11^, ^12^, ^13^, ^14^, ^15^, ^16^, ^17^, ^18^, ^19^, ^20^, ^21^, ^22^, ^23^, ^24^, ^25^, ^26^, ^27^, ^28^, ^29^, ^30^, ^31^, ^32^, ^33^, ^34^, ^35^, ^36^, ^37^, ^38^, ^39^, ^40^, ^41^, ^42^

Patient-specific TEVGs created by combining electrospinning with 3D-printed mandrels demonstrated feasibility in sheep models: all grafts remained patent without aneurysm formation or ectopic calcification at 6 months; nanofiber scaffold was nearly completely resorbed with mechanical properties similar to native inferior vena cava; organized SMC layer, ECM deposition, and endothelialization were observed; and significant positive correlation between wall thickness and CD68 macrophage infiltration was identified.^43^

### Melt electrowriting: Precision manufacturing

MEW represents a significant advance over conventional electrospinning by enabling precise control over fiber placement and architecture.^23^

MEW’s reproducibility and precision make it ideal for standardized manufacturing meeting regulatory requirements for batch-to-batch consistency – a critical consideration for clinical translation of peripheral vascular grafts requiring reliable performance.^23^

### 3D bioprinting and hybrid systems

3D bioprinting enables creation of complex, patient-specific geometries impossible with other methods.^1^, ^2^, ^3^, ^4^, ^5^, ^6^, ^7^, ^8^, ^9^, ^10^, ^11^, ^12^, ^13^, ^14^, ^15^, ^16^, ^17^, ^18^, ^19^, ^20^, ^21^, ^22^, ^23^, ^24^, ^25^, ^26^, ^27^, ^28^, ^29^, ^30^, ^31^, ^32^, ^33^, ^34^, ^35^, ^36^, ^37^, ^38^, ^39^, ^40^, ^41^, ^42^

Hybrid bioprinting-electrospinning systems combine the advantages of both technologies.^44^ These systems enable layered structures from electrospun fibers and cell-laden hydrogel, capability to produce tubular constructs with controlled architecture, and potential for incorporating multiple cell types in defined spatial arrangements.^44^

A blood vessel mimic created through hierarchical biofabrication demonstrated internal conduit of coelectrospun gelatin/PCL nanofibers (tunica intima); reinforcing layer of aligned PCL fibers (internal elastic membrane); bioprinted cell-laden hydrogel layers with SMCs and pericytes (tunica media/adventitia); semiautomated cellularization reducing production time to 6 days; and successful implantation in swine left pulmonary artery with patency at 5 weeks.^45^

### Multilayered manufacturing strategies

Multilayered architectures combining multiple fabrication methods have emerged as the most promising approach for clinical translation.^46^^,^^47^

A four-layered PCL-based graft combining electrospinning and four-axis printing achieved: inner layer with tightly aligned electrospun nanofibers supporting rapid endothelium formation, preventing leakage at >1100 mmHg; middle layers with circumferential electrospun nanofibers plus four-axis printed microfibers promoting human coronary artery SMC adhesion and infiltration; outer layer with randomly oriented electrospun nanofibers providing elasticity, toughness, and burst pressure resistance; and marine sulfated polysaccharide functionalization enhancing endothelialization and antithrombogenic properties.^46^

A cell-free multilayered scaffold combining 3D printing and electrospinning demonstrated in canine femoral artery model resistance to kinking and no blood seepage due to helical structure; high patency rate and remodeling ability at 6 months; and endothelialized lumen with SMC infiltration and ECM deposition.^47^ This canine femoral artery model is particularly relevant for peripheral vascular translation given its anatomical similarity to human infrainguinal vessels.

## Clinical translation experience: First-in-human studies

Despite the preclinical emphasis of the literature reviewed above, a number of tissue-engineered and regenerative small-diameter conduits have progressed to first-in-human evaluation over the past two decades; this experience, which spans several fabrication philosophies and centers worldwide, is summarized and classified by development stage in Table II.^48^, ^49^, ^50^, ^51^, ^52^, ^53^, ^54^, ^55^, ^56^, ^57^, ^58^, ^59^, ^60^ Completely biological conduits produced by sheet-based self-assembly (L’Heureux et al^48^, ^49^, ^50^) were among the first to reach patients, reported as a hemodialysis access conduit with patency of 78% at 1 month and 60% at 6 months, and subsequently as the first human use of an allogeneic version.^48^, ^49^, ^50^ Bioabsorbable, in situ-regenerating conduits (the Xeltis aXess graft) and precision-porous grafts based on the sphere-templated sphere-templated angiogenic regeneration biomaterial (Ratner and colleagues; Healionics STARgraft, NCT05729620) have since entered first-in-human dialysis-access trials, whereas cell-seeded biodegradable scaffolds (Shin’oka and Breuer) have served as low-pressure Fontan conduits in children.^55^, ^56^, ^57^ Decellularized fibrin-based grafts (Tranquillo) and electrospun PCL grafts (Kong) remain in preclinical and translational evaluation.^58^, ^59^, ^60^

**Table II:** First-in-human and translational experience with tissue-engineered and regenerative small-diameter vascular conduits

Platform (developer)	Origin	Construct type and principle	Application(s)	Furthest development stage	Selected evidence
Cytograft tissue-engineered blood vessel (L’Heureux, McAllister)	USA; Argentina, Poland	Completely biological, sheet-based self-assembled tissue (autologous and later allogeneic)	Arterial revascularization; hemodialysis access	Clinical (first-in-human)	48, 49, 50
Humacyte ATEV/HAV (Niklason)	USA	Decellularized bioengineered human vessel (vascular smooth muscle cells on a polyglycolic acid scaffold, bioreactor-cultured, then decellularized); off-the-shelf	Vascular trauma; peripheral arterial bypass; hemodialysis access	Clinical; FDA-approved for trauma (2024); pivotal trials	51, 52, 53, 54
Xeltis aXess	Europe (the Netherlands)	Bioabsorbable electrospun supramolecular polymer; in situ endogenous tissue restoration	Hemodialysis access	Clinical (first-in-human; EU pivotal)	55
Healionics STARgraft (Ratner; STAR biomaterial)	USA	Precision sphere-templated microporous (“STAR”) prohealing biomaterial graft	Hemodialysis access	Clinical (first-in-human; NCT05729620)	56
Shin’oka-Breuer tissue-engineered vascular graft	Japan; USA	Biodegradable scaffold seeded with autologous bone-marrow mononuclear cells	Pediatric congenital (Fontan) venous conduit	Clinical (first-in-human)	57
Tranquillo TRUE Tissue (Vascudyne)	USA	Decellularized, fibrin-based biologically engineered tissue tube	Arterial replacement; pediatric growth conduit	Translational (large animal)	58,59
Kong in situ-regeneration grafts (Nankai University)	China	Surface- and architecture-functionalized electrospun poly(ε-caprolactone); reinforced biotubes	Small-diameter arterial; arteriovenous grafting	Preclinical/translational (small and large animal)	60

*ATEV*, Acellular tissue engineered vessel; *FDA*, US Food and Drug Administration; *HAV*, human acellular vessel; *STAR*, sphere-templated angiogenic regeneration.

Development stage reflects the most advanced stage reported for each platform: preclinical (in vitro or small-animal), translational (large-animal or primate), or clinical (in humans).

The most extensive arterial experience has been accrued with the bioengineered acellular tissue engineered vessel (Niklason and colleagues), produced by culturing human vascular SMCs on a biodegradable polyglycolic acid scaffold in a pulsatile bioreactor and then decellularizing the construct to yield an off-the-shelf, nonimmunogenic 6-mm conduit that recellularizes with host cells after implantation.^51^ In December 2024, it became the first bioengineered vascular conduit to receive US Food and Drug Administration approval (as Symvess), for extremity arterial trauma when autologous vein is not feasible, and it remains the only such conduit to have completed pivotal-stage trials.^51^, ^52^, ^53^, ^54^ Of particular relevance to peripheral reconstruction, a phase II study demonstrated durable performance in above-the-knee femoropopliteal bypass,^52^ and an early single-center Mayo Clinic series reported encouraging results for infrainguinal, frequently tibial-level, bypass in CLTI without available saphenous vein: among 29 patients, limb salvage was 86% and secondary patency 71% at a median follow-up of approximately 9 months.^53^ Nevertheless, most first-in-human data derive from lower-pressure access or pediatric venous conduits; thrombosis, stenosis, and reintervention persist even in the most advanced arterial programs; and no tissue-engineered conduit has yet matched autologous vein in below-the-knee reconstruction—underscoring the need for preclinical designs that reflect the clinical arterial environment, as discussed further.

## Designing preclinical studies that predict clinical outcomes

The translation of promising small-diameter grafts from bench to bedside has been hampered by inadequate preclinical study designs that fail to predict clinical performance. A meta-analysis of 68 preclinical trials revealed that median graft length (5 cm) and median follow-up (56 days) are inadequate for direct clinical translation.^39^ For peripheral vascular applications where bypass grafts often exceed 30 cm and patency must be maintained for years, these limitations are particularly consequential.

### The translational gap

A landmark analysis identified a fundamental problem: inappropriate animal models addressing scientific questions that miss clinical relevance have dominated vascular graft research for decades.^61^ The vast majority of investigators unintentionally studied transanastomotic endothelialization rather than transmural or blood-borne endothelialization.^61^

This distinction is critical because in patients, transanastomotic endothelialization covers only the immediate perianastomotic region of sometimes very long prostheses, making it clinically irrelevant.^61^ By selecting animal models that achieve rapid transanastomotic endothelialization (dogs and pigs), researchers obscured the most significant barrier to clinical success.^61^

In addition, most evaluation studies are performed in healthy animals that cannot represent the clinical situation.^62^ Cardiovascular grafting procedures are predominantly performed in elderly patients with comorbidities – diabetes, hyperlipidemia, chronic kidney disease – yet young, healthy animals are preferred for preclinical testing.^62^

A hypercholesterolemia rat model demonstrated this problem: PCL grafts showed lower patency compared with healthy controls; significant neo-IH and abundant collagen deposition occurred; slower endothelialization was observed; and endothelial cells from hypercholesterolemic aortas showed impaired proliferation, migration, and adhesion under both static and pulsatile flow conditions.^62^ DNA microarray studies revealed downregulation of genes involved in EC proliferation, apoptosis, and metabolism.^62^ These findings emphasize that future preclinical evaluation should incorporate disease animal models reflecting clinical patient populations.^62^

### Species selection for peripheral vascular translation

Species selection must consider differences in coagulation and hemostatic profiles.^63^^,^^64^ Nonhuman primates most closely replicate human physiology, whereas sheep provide the most comparable coagulation profiles based on rotational thromboelastometry (ROTEM) analysis.^64^ Pigs offer anatomical similarity and established disease models but may overestimate performance due to rapid endothelialization and neo-IH.^65^, ^66^, ^67^, ^68^

Smaller animal models remain useful for early-stage evaluation. Rats enable mechanistic studies but have limited translational relevance due to vessel size.^69^^,^^70^ Rabbits provide intermediate validation with human-like hemostasis and compatibility with clinically relevant anastomotic techniques.^71^

Overall, large animal models – particularly sheep and pigs – are preferred for translational studies, whereas nonhuman primates remain the gold standard for predictive validation.^72^^,^^73^

### Critical study design elements

Based on the evidence, specific recommendations can be made for preclinical studies intended to support peripheral vascular translation:

Graft length and diameter must reflect clinical reality. Clinical infrainguinal grafts often exceed 30 cm, yet preclinical studies use median lengths of only 5 cm.^39^ Recommendations include minimum graft length of 10 cm for large animal studies and diameter matching intended clinical application (4-6 mm for femoropopliteal, 3-4 mm for infrapopliteal).

Follow-up duration must extend beyond current norms. Median follow-up of 56 days cannot predict long-term patency in clinical applications.^39^ Minimum follow-up should be 6 months, preferably 12 to 24 months for large animal studies.

Anastomotic technique should match clinical practice. Clinical vascular grafting uses end-to-side surgical methods, but researchers primarily use end-to-end techniques, affecting flow patterns, stress distribution, and IH development.^71^ Studies demonstrate that end-to-side anastomosis is feasible even in small animals and should be implemented to improve translational validity.^71^

Control groups should include autologous vessel control for optimal performance benchmark and ePTFE control for comparison to current clinical standard.^65^^,^^69^, ^70^, ^71^

Disease models should be incorporated to reflect clinical patient populations, including hypercholesterolemia, diabetes, and aged animals rather than juvenile subjects.^62^, ^63^, ^64^, ^65^, ^66^, ^67^, ^68^, ^69^, ^70^, ^71^

## Current state-of-the-art performance and future directions

Recent preclinical studies have achieved impressive benchmarks approaching clinical viability for peripheral vascular applications.^19^, ^20^, ^21^, ^22^, ^23^, ^24^, ^25^^,^^38^

Poly(vinyl alcohol)/dextran hydrogel grafts with MSC-based therapies demonstrated similar or higher patency rates compared with ePTFE controls in an ovine model over 24 weeks, with reduced fibrosis, endothelial cells detected at graft-artery transitions, and maintained systolic and diastolic laminar blood flow velocities.^38^

However, these results must be interpreted cautiously: median graft length (5 cm) and median follow-up (56 days) remain inadequate for direct clinical translation to peripheral vascular applications.^39^ The key limitations of current preclinical models and their implications for clinical translation are summarized in Table III.

**Table III:** Limitations of current preclinical models and implications for clinical translation of small-diameter vascular grafts

Parameters	Typical preclinical studies	Clinical reality	Implication for translation
Graft length	Short grafts (∼5 cm)	Often long grafts (>30 cm)	Underestimation of thrombosis and intimal hyperplasia risk
Follow-up duration	Short term (∼56 days)	Long term (years)	Limited ability to assess durability and late failure mechanisms
Animal model	Healthy vessels	Diseased arteries (eg, PAD and diabetes)	Overestimation of graft performance
Hemodynamic environment	Controlled conditions	Complex, variable flow patterns	Limited prediction of real-world performance
End points	Patency, histology	Patency, limb salvage, reintervention	Incomplete assessment of clinical outcomes

*PAD*, Peripheral arterial disease.

### Future research priorities

The field is converging on several priorities for clinical translation in peripheral vascular surgery:

Hybrid biomaterial systems combining synthetic mechanical strength with natural biological activity represent the most promising approach, with cardiac ECM, silk fibroin, and decellularized matrices showing particular promise.^20^, ^21^, ^22^, ^23^, ^24^, ^25^, ^26^, ^27^, ^28^, ^29^, ^30^, ^31^, ^32^, ^33^^,^^38^^,^^39^

Multilayered architectures with designated functions for each layer address multiple simultaneous requirements for success—an approach that has produced the most impressive preclinical results to date.^8^, ^9^, ^10^, ^11^^,^^14^, ^15^, ^16^, ^17^, ^18^, ^19^, ^20^

Precision manufacturing through MEW and hybrid electrospinning/3D printing enables reproducible, scalable production meeting regulatory requirements for batch-to-batch consistency.^23^, ^24^, ^25^, ^26^, ^27^, ^28^, ^29^, ^30^, ^31^, ^32^, ^33^, ^34^, ^35^, ^36^, ^37^, ^38^, ^39^, ^40^, ^41^, ^42^, ^43^

Bioactive molecule incorporation addresses both acute thrombosis and chronic IH through sustained local delivery of heparin, VEGF, C-type natriuretic peptide, and NO donors.^17^, ^18^, ^19^, ^20^, ^21^, ^22^, ^23^, ^24^, ^25^, ^26^, ^27^, ^28^, ^29^, ^30^, ^31^^,^^38^^,^^39^

Immunomodulation strategies promoting M2 macrophage polarization reduce inflammation-driven complications while supporting constructive remodeling.^18^, ^19^, ^20^, ^21^, ^22^, ^23^, ^24^, ^25^, ^26^, ^27^, ^28^, ^29^, ^30^, ^31^^,^^38^^,^^39^

Improved preclinical study design incorporating clinically relevant graft lengths, disease models, extended follow-up, and appropriate animal species selection will be essential for predicting clinical outcomes in peripheral vascular applications.^26^, ^27^, ^28^, ^29^, ^30^, ^31^, ^32^, ^33^, ^34^, ^35^, ^36^, ^37^^,^^39^, ^40^, ^41^, ^42^, ^43^, ^44^, ^45^, ^46^, ^47^, ^48^, ^49^, ^50^, ^51^, ^52^, ^53^, ^54^, ^55^, ^56^, ^57^, ^58^, ^59^, ^60^, ^61^, ^62^

## Conclusions

The small-diameter vascular graft remains a major unresolved challenge in peripheral vascular surgery. Four decades of research have elucidated the mechanisms of failure – thrombosis, IH, compliance mismatch, and incomplete endothelialization – while generating increasingly sophisticated solutions.

For vascular surgeons managing patients with CLTI who lack suitable autologous conduits, current synthetic options remain inadequate, with patency rates of 25% to 50% at 5 years for below-the-knee applications and up to 20% of patients experiencing thrombosis within 6 months. The clinical consequences – graft failure, reintervention, and limb loss – underscore the urgency of developing improved alternatives.

Recent advances in multilayered scaffold design, hybrid biomaterials, precision manufacturing, and immunomodulatory strategies have produced preclinical results approaching clinical viability, with multiple studies reporting 100% patency, complete endothelialization, and absence of IH. However, these promising findings must be validated through rigorous preclinical studies using clinically relevant graft dimensions appropriate for infrainguinal bypass, animal models incorporating disease states reflecting actual patient populations, and extended follow-up periods.

The clear benchmarks for success (>80% patency at 5 years, comparable to autologous vein) and well-defined failure modes make small-diameter grafts an ideal focus for systematic translational research. The convergence of advanced biomaterials, precision manufacturing, mechanistic understanding, and improved preclinical methodology suggests that clinically viable small-diameter TEVGs for peripheral vascular reconstruction may be achievable within the next decade.

Success will require simultaneous optimization of antithrombogenicity, endothelialization, compliance matching, and inflammation control—a challenge that multilayered, multifunctional designs are increasingly capable of meeting. For vascular surgeons, this progress offers hope that the substantial population of patients lacking suitable autologous conduits may eventually have access to reliable alternatives, improving limb salvage rates and quality of life for patients with PAD.

## Author Contributions

Conception and design: MS, ES, CN

Analysis and interpretation: MS, LB, LO, JC, PD, BT, MF

Data collection: Not applicable

Writing the article: MS, LB, LO, JC, PD, BT

Critical revision of the article: MS, ES, CN, MF

Final approval of the article: MS, LB, LO, JC, PD, BT, ES, CN, MF

Statistical analysis: Not applicable

Obtained funding: Not applicable

Overall responsibility: MS

## Funding

None.

## Disclosures

None.
